# Clinical features and outcomes of COVID-19 admissions in a population with a high prevalence of HIV and tuberculosis: a multicentre cohort study

**DOI:** 10.1186/s12879-022-07519-8

**Published:** 2022-06-20

**Authors:** Arifa Parker, Linda Boloko, Muhammad S. Moolla, Nabilah Ebrahim, Birhanu T. Ayele, Alistair G. B. Broadhurst, Boitumelo Mashigo, Gideon Titus, Timothy de Wet, Nicholas Boliter, Michael-Jon Rosslee, Nectarios Papavarnavas, Riezaah Abrahams, Marc Mendelson, Sipho Dlamini, Jantjie J. Taljaard, Hans W. Prozesky, Abdurasiet Mowlana, Abraham J. Viljoen, Neshaad Schrueder, Brian W. Allwood, Usha Lalla, Joel A. Dave, Greg Calligaro, Dion Levin, Deborah Maughan, Ntobeko A. B. Ntusi, Peter S. Nyasulu, Graeme Meintjes, Coenraad F. N. Koegelenberg, Ayanda T. Mnguni, Sean Wasserman

**Affiliations:** 1 Division of General Medicine, Department of Medicine, Stellenbosch University and Tygerberg Hospital, Cape Town, South Africa; 2 Department of Medicine, University of Cape Town, Cape Town, South Africa; 3 Department of Medicine, Khayelitsha District Hospital, Cape Town, South Africa; 4 Division of Epidemiology and Biostatistics, Department of Global Health, Stellenbosch University, Cape Town, South Africa; 5 Division of Infectious Diseases and HIV Medicine, Department of Medicine, University of Cape Town, Cape Town, South Africa; 6 Division of Infectious Diseases, Department of Medicine, Stellenbosch University and Tygerberg Hospital, Cape Town, South Africa; 7 Division of Pulmonology, Department of Medicine, Stellenbosch University and Tygerberg Hospital, Cape Town, South Africa; 8 Present Address: Extramural Unit on Intersection on Noncommunicable Diseases and Infectious Diseases, South African Medical Research Council, Cape Town, South Africa; 9 Institute for Infectious Disease and Molecular Medicine, Wellcome Centre for Infectious Diseases Research in Africa, University of Cape Town, Cape Town, South Africa

**Keywords:** HIV, COVID-19, Tuberculosis, Obesity

## Abstract

**Background:**

There is still a paucity of evidence on the outcomes of coronavirus disease 2019 (COVID-19) among people living with human immunodeficiency virus (PWH) and those co-infected with tuberculosis (TB), particularly in areas where these conditions are common. We describe the clinical features, laboratory findings and outcome of hospitalised PWH and human immunodeficiency virus (HIV)-uninfected COVID-19 patients as well as those co-infected with tuberculosis (TB).

**Methods:**

We conducted a multicentre cohort study across three hospitals in Cape Town, South Africa. All adults requiring hospitalisation with confirmed COVID-19 pneumonia from March to July 2020 were analysed.

**Results:**

PWH comprised 270 (19%) of 1434 admissions. There were 47 patients with active tuberculosis (3.3%), of whom 29 (62%) were PWH. Three-hundred and seventy-three patients (26%) died. The mortality in PWH (n = 71, 26%) and HIV-uninfected patients (n = 296, 25%) was comparable. In patients with TB, PWH had a higher mortality than HIV-uninfected patients (n = 11, 38% vs n = 3, 20%; p = 0.001). In multivariable survival analysis a higher risk of death was associated with older age (Adjusted Hazard Ratio (AHR) 1.03 95%CI 1.02–1.03, p < 0.001), male sex (AHR1.38 (95%CI 1.12–1.72, p = 0.003) and being “overweight or obese” (AHR 1.30 95%CI 1.03–1.61 p = 0.024). HIV (AHR 1.28 95%CI 0.95–1.72, p 0.11) and active TB (AHR 1.50 95%CI 0.84–2.67, p = 0.17) were not independently associated with increased risk of COVID-19 death. Risk factors for inpatient mortality in PWH included CD4 cell count < 200 cells/mm^3^, higher admission oxygen requirements, absolute white cell counts, neutrophil/lymphocyte ratios, C-reactive protein, and creatinine levels.

**Conclusion:**

In a population with high prevalence of HIV and TB, being overweight/obese was associated with increased risk of mortality in COVID-19 hospital admissions, emphasising the need for public health interventions in this patient population.

## Introduction

There is still a paucity of evidence on the outcomes of coronavirus disease 2019 (COVID-19) among people living with human immunodeficiency virus (PWH) and those co-infected with tuberculosis (TB), particularly in areas where these conditions are common. Southern Africa has the highest human immunodeficiency virus (HIV) prevalence [1] and one of the highest incidence rates of TB globally. [2] With the added high local burden of non-communicable diseases (NCDs) [3, 4] our study population is uniquely suited to explore the interplay between the effects of these colliding pandemics on the epidemiology of COVID-19. [5] The first COVID-19 case in South Africa was reported in March 2020, with the peak of the first wave occurring in July 2020. [6]

HIV is associated with reduced T-cell mediated and humoral immune responses increasing host susceptibility to opportunistic infections. [7] Severe COVID-19 is associated with elevated pro-inflammatory cytokines and innate immune responses, and it has been hypothesized that the immune deficiency seen in HIV may ameliorate severe acute respiratory syndrome coronavirus 2 (SARS-CoV-2) pathology. [8] It has also been suggested that antiretroviral therapy (ART) may have some activity against SARS-CoV-2. [9] Emerging evidence suggests that HIV and TB co-infection is associated with a reduced T cell and humoral response to SARS-CoV-2, and that SARS-CoV-2 itself reduces CD4 T cell lymphocyte levels, which may be mechanisms for poor clinical outcomes [10].

Comorbidities, including hypertension, diabetes mellitus and obesity are known to be associated with severe COVID-19 and poor outcomes [11–13]. These comorbidities are associated with COVID-19 admission in PWH and some studies have suggested that, similar to HIV-uninfected patients, a higher burden of these NCDs may be driving COVID-19 mortality in PWH [7, 14, 15].

A systemic review of earlier studies done globally [7] did not show an increased mortality risk in PWH, but the outcomes in these studies may have been limited by small sample sizes. In contrast, larger studies have since demonstrated increased mortality risk in PWH [16–18]. While a large South African population cohort study demonstrated an increased risk of COVID-19 mortality in both PWH and TB [16], this study did not have access to data usually present in patient folders, such as prevalence of obesity in COVID-19 admissions, and this may have influenced the results. There is a paucity of outcome data for patients with TB and COVID-19. Very few studies, with small sample sizes, have investigated mortality in patients with active TB and COVID-19 [19–21].

The primary aim of this study was to describe the clinical features, laboratory findings and outcome of PWH and HIV-uninfected COVID-19 hospitalised patients, and specifically to determine whether an independent association of HIV, TB and other comorbidities exist, with in-hospital mortality in all COVID-19 admissions. The secondary aims were to identify predictors of inpatient mortality in PWH admitted to hospital with COVID-19.

## Methods

### Study design and population

This observational study included all patients, 18 years and older with COVID-19 as confirmed by a positive SARS-CoV-2 reverse transcriptase polymerase chain reaction (RT-PCR) result requiring hospital admission from the 1st of March 2020 until the 31st of July 2020. Data was captured prospectively at Tygerberg Hospital (TBH) and Khayelitsha District Hospital (KDH), and retrospectively at Groote Schuur Hospital (GSH) from a prospective application-based registry. Patients were followed up until hospital admission course (either discharge or death) was completed. Patients with missing outcome data were excluded. Our study cohort was previously included in a population-based cohort study described elsewhere [16].

### Settings

GSH (893 beds) and TBH (1380 beds) are urban tertiary academic referral hospitals which serve the greater Western Cape population. KDH is a 330-bed district hospital in Khayelitsha, a peri-urban township community with a very high burden of HIV and TB. Ethical approval with waiver of informed consent for this study was obtained from the Health Research Ethics Committee (HREC) of the University of Cape Town (HREC REF: 285-2020) for GSH and the Stellenbosch University HREC for TBH (N20/04/002_COVID-19) and KDH (N20/05/020_COVID-19). TBH provides tertiary level care services for KDH, which includes the transfer of patients requiring higher level of care (non-invasive or invasive ventilation) to TBH. These duplicate admissions of transferred patients were merged as a single hospital admission.

### Data collection

We collected demographic, clinical and laboratory data. Clinical data included symptoms on admission to hospital, the presence of comorbidities including HIV (a diagnostic and confirmatory fourth-generation HIV chemiluminescence-immunoassay was routinely performed on all patients for whom the HIV status was not known), hypertension, diabetes, overweight/obesity (defined by a clinician’s impression of overweight/obesity), any underlying cardiac and chronic kidney diseases (as recorded in the clinical notes), and active or previous TB. Active TB was defined as any patient who was prescribed TB treatment by the patient care team (either commenced prior to or during admission). We also captured a measure of pre-morbid functional status utilising a clinical frailty scale (CFS), which graded frailty on a score ranging from 1 (very fit) to 9 (terminally ill) [22].

Laboratory values collected included the partial pressure of arterial oxygen (in mmHg) to fraction of inspired oxygen (P:F ratio), the white blood cell (WBC), the neutrophil to lymphocyte ratio (N:L ratio), serum creatinine and the C-Reactive Protein (CRP). CD4 cell counts and HIV viral loads in PWH were captured if they were performed within a year of admission or on admission. Investigations and management were performed as part of routine care and thus at the discretion of treating clinicians.

### General patient management

During the first wave, all COVID-19 infected patients, including PWH and TB, were routinely managed with supplemental oxygen, awake proning, and enoxaparin. Prior to the release of the Recovery trial results [23], steroids were prescribed on an “ad hoc” basis, at the discretion of the patient care team, and was only adopted as routine standard of care thereafter [24].

### Statistical analysis

We analysed demographic and clinical characteristics as well as pre-existing comorbidities at baseline and compared them between the two groups ‘HIV-uninfected and PWH’. We further computed the frequencies and proportions of these characteristics and compared the differences between these groups using X^2^ test of independence. We used the Wilcoxon rank-sum tests to compare between mild/moderate and severe/critical cases using medians parameter values. We conducted a univariable logistic regression analysis to assess individual factors associated with in-hospital mortality. To assess independent factors associated with mortality we included all factors with a p < 0.1 in the univariable model into a stepwise forward selection multivariable logistic regression model for crude and adjusted Odds Ratios, and COX proportional model to calculate crude and adjusted Hazard Ratios. We used the Hosmer and Lemeshow’s goodness-of-fit test to assess how well the model fitted the data. We conducted stratified analysis to evaluate factors associated with mortality among PWH. We determined the relationship between specific exposure factors and mortality using Odds Ratios, Risk Ratios, and corresponding 95% Confidence Intervals. Factors with p < 0.05 were considered significantly associated with mortality. All analyses were performed using Stata software version 16.1 (College Station, TX, USA).

## Results

**Fig. 1:**
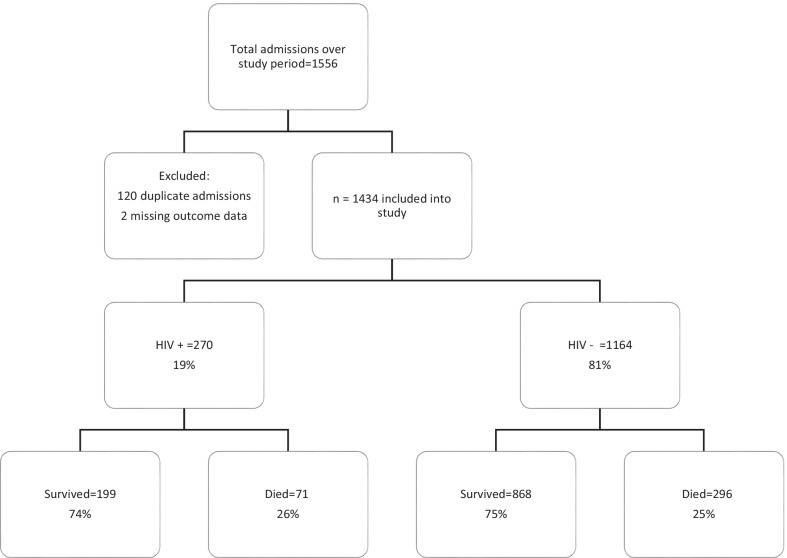
Consort diagram: distribution of study participants

There were 1556 admissions across the three study sites during the enrolment period. Of these GSH, TBH and KDH had 571, 597 and 388 admissions, respectively. A total of 1434 patients were included in the final analysis, of whom 270 (19%) were PWH (Fig. 1).

The baseline characteristics and indices of severity of PWH and HIV-uninfected patients are summarised in Table 1. There were more females than males in both the overall cohort (n = 817, 57%; p < 0.001) and in PWH (n = 183, 68%). PWH were younger [median age 46 years (interquartile range (IQR) 39–52 vs 54 IQR 43–65], p < 0.001]. The most common symptoms in both PWH and HIV-uninfected patients on admission were cough, shortness of breath and fever. PWH were more likely to have diarrhoea (p < 0.001) and less likely to have anosmia (p = 0.03).

**Table 1:** Baseline clinical characteristics, laboratory data and indices of severity of People with HIV (PWH) and HIV-uninfected patients

Parameter (IQR)	Reference value	n	HIV-uninfected (N = 1164)	PWH (N = 270)	*p*-value
Demographics*					
Age (years), median (IQR**)			54 (43–65)	46 (39–52)	< 0.001
Male, *n* (%)			530 (45.5%)	85 (31.7%)	< 0.001
Female, *n* (%)			634 (54.5%)	183 (68.3%)	
Presenting symptoms*, n (%)					
Cough			787 (67.6%)	195 (72.2%)	0.14
Dyspnoea			835 (71.7%)	198 (73.3%)	0.60
Fever			507 (43.6%)	122 (45.2%)	0.63
Sore throat			197 (16.9%)	36 (13.3%)	0.15
Anosmia			97 (8.3%)	12 (4.5%)	0.03
Diarrhoea			91 (7.8%)	40 (14.8%)	< 0.001
Myalgia			259 (22.3%)	50 (18.5%)	0.18
Comorbidities*, n (%)					
Hypertension			612 (52.6%)	103 (38.1%)	< 0.001
Diabetes mellitus			463 (39.8%)	73 (27.0%)	< 0.001
Overweight / obese			486 (41.8%)	79 (29.3%)	< 0.001
Cardiac disease			92 (7.9%)	9 (3.3%)	.008
Current Tuberculosis			18 (1.5%)	29 (10.7%)	< 0.001
Past Tuberculosis			40 (3.4%)	37 (13.7%)	< 0.001
Chronic kidney disease			86 (7.4%)	21 (7.8%)	0.79
Frailty score*, n (%)					0.13
Well	(1–3)		541 (71.6%)	85 (75.9%)	
Mild to moderate impairment	(4–6)		193 (25,5%)	26 (23.2%)	
Severe impairment	(7–9)		22 (2.9%)	1 (0.9%)	
*Severity indices*					
SaO_2_ (%)	93–97%	1251	93 (87–96)	93 (87–97)	0.40
PaO_2_ (kPa)	10.5–13.5	765	8.2 (6.4–10.8)	8.1 (6.3–11.2)	0.67
P:F ratio	≥ 400	727	189.3 (95.8–303.7)	228.9 (128.6–432.8)	0.019
WBC (× 10^9^ /L)	3.92–10.40	1395	8.23 (6.23–11)	8.9 (6.4–12.1)	0.09
N:L ratio	1–3	1153	4.91 (2.95–7.79)	4.69 (2.75–7.64)	0.34
CRP (mg/L)	< 10	1095	121 (56.5–206)	165.5 (94.8–270)	< 0.001
Creatinine (μmol/L)	64–104	1375	80 (62–110.5)	73 (57–107.3)	0.033
Haemoglobin (g/dL)	13.0–17.0	1384	13.1 (11.8–14.3)	12.6 (10.58–13.7)	< 0.001
Neutrophils (× 10^9^/L)	1.60–6.98	1148	6.34 (4.37–9.12)	6.59 (4.18–9.45)	0.96
Lymphocytes (× 10^9^/L)	1.40–4.20	1153	1.31 (0.92–1.85)	1.42 (0.95–1.92)	0.14
Platelets (× 10^9^/L)	171–388	1360	248 (196–324)	269 (208.5–361)	< 0.001
D-dimer (mg/L)	0.00–0.25	430	0.67 (0.38–1.86)	0.79 (0.35–2.50)	0.53

^*^Data available for n = 1434 patients, except Sex and Frailty score where data were available for n = 1432 and n = 868 patients respectively. ** IQR = interquartile range. SaO2 = oxygen saturation; PaO2 = arterial partial pressure of oxygen; FiO2 = fraction of inspired oxygen; P:F ratio = partial pressure of arterial oxygen to fraction of inspired oxygen; WBC = white blood cell; N:L ratio = neutrophil to lymphocyte ratio

Active TB was present in 47 patients (3.3%). Active TB (n = 29, 10.7%; p < 0.001) or a prior history of TB (n = 37, 13.7%; p < 0.001) was more prevalent in PWH than HIV-uninfected patients. PWH infected with TB had higher mortality than HIV-uninfected patients with TB (n = 11, 38% vs n = 3, 20%; p = 0.001).

In total, 373 (26%) patients died, including 71 deaths in PWH (26%) and 296 deaths in HIV-negative patients (25%). On multivariable logistic regression analysis, HIV was found to be an independent predictor of mortality [adjusted odds ratio (AOR) 1.56, 95% confidence interval (95%CI) 1.11–2.2, p = 0.011], along with older age (AOR 1.04 per year 95%CI 1.03–1.05, p < 0.001), male sex (AOR 1.62 95%CI 1.25–2.08, p < 0.001) and being ‘overweight or obese’ (AOR 1.51 95%CI 1.16–1.96 p = 0.002). Patients with active TB had an increased odds of mortality (AOR 2.01 95%CI 1.01–4.02, p = 0.049). A past history of TB was not predictive of outcome (AOR 1.62 95%CI 0.96–2.72, p = 0.07). While diabetes and hypertension were associated with mortality in unadjusted analysis, this was not found to be statistically significant when adjusting for potential confounders (Table 2). On Cox regression analysis, a higher risk of death was associated with older age (Adjusted hazard ratio (AHR) 1.03 95%CI 1.02–1.03, p < 0.001), male sex (AHR1.38 (95%CI 1.12–1.72, p = 0.003) and being “overweight or obese” (AHR 1.30 95%CI 1.03–1.61 p = 0.024) (Table 2).

**Table 2:** Crude and adjusted measures of odds ratio (OR) and hazard ratio (HR) for mortality in all patients

	Unadjusted OR (CI)	p-value	Adjusted OR (CI)	p-value	Unadjusted HR (CI)	p-value	Adjusted HR (CI)	p-value
Older Age*	1.04 (1.03–1.05)	< 0.001	1.04 (1.03–1.05)	< 0.001	1.02 (1.02–1.03)	< 0.001	1.03 (1.02–1.04)	< 0.001
Male sex	1.45 (1.14–1.84)	0.002	1.62 (1.26–2.08)	< 0.001	1.27 (1.03–1.57)	0.025	1.38 (1.12–1.72)	0.003
Overweight/obesity	1.23 (0.98–1.58)	0.07	1.51(1.16–1.96)	0.002	1.11 (0.90–1.37)	0.32	1.29 (1.03–1.61)	0.024
HIV	1.05 (0.77–1.41)	0.77	1.56 (1.11–2.21)	0.011	1.07 (0.82–1.40)	0.60	1.28 (0.95–1.72)	0.11
Diabetes	1.51 (1.18–1.92)	0.001	1.14 (0.86–1.49)	0.359	1.21 (0.98–1.49)	0.08	0.99 (0.79–1.24)	0.95
Hypertension	1.81 (1.42–2.31)	< 0.001	1.14 (0.85–1.53)	0.388	1.50 (1.21–1.86)	< 0.001	1.01 (0.99–1.01)	0.091
Active TB	1.24 (0.66–2.35)	0.51	2.01 (1.01–4.02)	0.049	1.03 (0.59–1.79)	0.92	1.50 (0.84–2.67)	0.170
Previous TB	1.52 (0.93–2.47)	0.09	1.62 (0.96–2.72)	0.07	1.12 (0.75–1.69)	0.57	1.18 (0.77–1.82)	0.430

*CI* confidence interval, *HIV* human immunodeficiency viruses, *TB* tuberculosis

^*^Per one year increase in age

In PWH, 214 (79%) were on ART and 204 (78%) had HIV viral loads of < 1000 copies/mL (Tables 3 and 4). The CD4 cell count was ≥ 200 cells/mm^3^ in 147/222 (66%) patients. A CD4 count of < 200 cells/mm^3^, higher neutrophil and lower lymphocyte counts, and indices of illness severity (higher FiO_2_ requirement, WBC, N:L ratio, CRP and creatinine) were predictive of inpatient mortality. HIV viral load level and ART usage were not associated with patient outcomes. PWH had a non-significant longer median length of stay in hospital [27 (15–39) vs. 20 (17–23) days] compared to HIV-uninfected patients.

**Table 3:** Baseline clinical predictors of outcome in among People with HIV (PWH, n = 270)

Parameter	Survivors n = 199 (73.7%)	Non-survivors n = 71 (26.3%)	RR (95% CI)	P value
On ART (n = 214)	153 (71.5%)	61 (28.5%)	0.90 (0.79–1.01)	0.11
Not on ART (n = 56)	46 (82.1%)	10 (17.9%)		
Current TB (n = 29)	18 (62.1%)	11 (37.9%)	1.52 (0.91–2.55)	0.13
Previous TB (n = 37)	25 (67.6%)	12 (32.3%)	1.28 (0.77–2.14)	0.36
Overweight (n = 79)	59 (74.7%)	20 (25.3%)	1.95 (.0.61–1.48)	0.81
Diabetes (n = 73)	52 (71.2%)	21 (28.8%)	1.13 (0.73–1.75)	0.57
Hypertension (n = 103)	71 (68.9%)	32 (31.1%)	1.33 (0.89–1.98)	0.16

*ART*  antiretroviral treatment, *TB*  tuberculosis, *RR* risk ratio

**Table 4:** Baseline laboratory predictors of outcome in among People with HIV (PWH, n = 270)

Parameter*	Survivors n = 199 (73.7%)	Non-survivors n = 71 (26.3%)	COR** (95% CI)	P value
CD4 (n = 222)			1.92 (1.03–3.56)	0.04
≥ 200 (n = 147)	116 (78.9%)	31 (21.1%)		
< 200 (n = 75)	49 (65.3%)	26 (34.7%)		
HIV viral load (n = 262)			1.70 (0.82–3.5)	0.15
< 1000 (n = 204)	146 (71.6%)	58 (28.4%)		
> 1000 (n = 58)	47(81.0%)	11 (19.0%)		
PaO_2_ (kPa) (n = 150)	8.8 (6.5–11.7)	7.2 (5.9–10.5)	0.97 (0.91–1.04)	0.39
FiO_2_ (%) (n = 178)	0.21 (0.21–0.4)	0.4 (0.21–0.8)	5.34 (1.53–18.63)	0.01
P:F ratio (n = 147)	264.3 (159.9–344.2)	172.5 (84–242.8)	0.997 (0.983–1.010)	0.64
WBC (n = 269)	8.3 (6.12–11.1)	10.23 (8.4–14.0)	1.093 (1.036–1.154)	0.001
N:L ratio (n = 227)	4.0 (1.45–6.7)	6.6 (4.2–11.8)	1.12 (1.06–1.19)	< 0.001
CRP (n = 214)	145 (77–240.5)	247 (151.5–336)	1.005 (1.002–1.007)	< 0.001
Creatinine (μmol/L) (n = 266)	71 (53.25–96.75)	89.5 (63.75–275)	1.003 (1.001–1.004)	< 0.001
Haemoglobin (g/dL) (n = 266)	12.7 (10.8–13.8)	12.3 (9.3–13.5)	1.021 (0.99–1.05)	0.15
HbA1c (%) (n = 81)	7.5 (6.8–8.4)	7.0 (6.0–8.3)	0.98 (0.95–1.02)	0.42
Neutrophils (× 10^9^/L) (n = 225)	5.74 (3.95–8.34)	8.25 (6.35–11.65)	1.102 (1.04–1.17)	0.002
Lymphocytes (× 10^9^/L) (n = 227)	1.57 (1.08–2.03)	1.18 (0.77–1.78)	0.51 (0.31–0.8)	0.004
Platelets (× 10^9^/L) (n = 265)	268 (212.3–357.75)	276 (197–367)		0.289
D-dimer (mg/L) (n = 52)	0.61 (0.35–2.36)	0.88 (0.54–6.81)	1.15 (0.98–1.33)	0.78

*SaO*_*2*_ oxygen saturation, *PaO*_*2*_  arterial partial pressure of oxygen, *FiO*_*2*_ fraction of inspired oxygen, *P:F ratio*  partial pressure of arterial oxygen to fraction of inspired oxygen, *WBC*  white blood cell, *N/L ratio*  neutrophil to lymphocyte ratio, *HbA1c*  glycated haemoglobin

*Median (IQR) unless otherwise stated

**COR, crude odds ratio

## Discussion

In this multicentre cohort of patients hospitalised with COVID-19 during the first wave in South Africa, the crude mortality rate was similar between PWH and HIV-uninfected patients. On logistic regression analysis,  after adjusting for potential confounders (older age, male sex, overweight/obesity, hypertension, diabetes, active and previous tuberculosis), we found that HIV, active TB, older age, male sex and being ‘overweight or obese’ were associated with increased odds of death in COVID-19 admissions. However, when adjusting for time to event (survival or death), only older age, male gender and being ‘overweight or obese’, and not HIV or active TB, were independently associated with an increased risk of death in our study.

Earlier studies have suggested that PWH did not have increased risk of severe COVID-19 or COVID-19 mortality [1, 4, 8], but these studies were limited by smaller sample sizes. Our study population was also included in a South African population based cohort study by Boulle et al., and on logistic regression mirrored their finding that HIV and active TB was independently associated with death in hospitalised COVID-19 patients [16]. However, a limitation to that study was that clinical data such as weight was not available and could therefore not be included in adjusted analyses. Our study included clinical data captured at the bedside and found that being ‘overweight or obese’ was associated with a 30% increase in the risk of COVID-19 mortality in adjusted survival analysis.

Many earlier studies have found that comorbidities including hypertension, diabetes mellitus and heart disease were associated with death due to COVID-19, but did not include obesity as a risk factor [16, 25]. Obesity has since emerged as a key risk factor for severe COVID-19 and mortality [26, 27]. Similarly, our study found that diabetes and hypertension were associated with mortality in unadjusted analysis, but not when adjusted for other confounders, like obesity and HIV. Our data suggests that obesity, prevalent in the patients with hypertension or diabetes, may be driving mortality in patients with these comorbidities. A possible explanation for this is the fact that the receptors, which enable SARS-CoV-2 entry into cells, angiotensin-converting-enzyme 2 (ACE 2) and in theory dipeptidyl peptidase 4 (DPP4), have increased expression on adipocytes of obese people [28]. There is a suggestion that the adipocyte could be a viral reservoir resulting in an increased SARS-CoV2 viral load. This, coupled with impaired T cell mediated immune responses seen in obesity, may result in the cytokine release syndrome resulting in COVID-19 mortality [29]. It remains unclear whether obesity may be influencing COVID-19 related mortality in PWH, since formal body mass indices (BMI) are not routinely measured in many studies, and this comorbidity may therefore be underrepresented.

This was a relatively well-controlled HIV cohort with the majority of PWH on ART and with HIV viral loads of < 1000 copies/mL in approximately three quarters. A third of patients however had CD4 counts of < 200 cells/mm^3^. Caution should be applied in the interpretation of this CD4 count association, as the CD4 status may not reflect baseline immune status as a number of CD4 counts were performed on the acute COVID-19 admission, where both acute illnesses and SARS-CoV-2 infection are documented to decrease circulating lymphocyte populations [30].

Previous studies have failed to show differences in laboratory parameters of disease severity between PWH and HIV-uninfected patients [13, 31]. In our study PWH had significantly higher CRP levels and lower haemoglobin and creatinine levels suggesting underlying chronic disease and immune activation. The CRP, a non-specific marker of inflammation, may be elevated due to a sustained immune response from HIV infection itself [32], or due to co-infections, such as TB or pneumonia [33]. In a study of patients with appendicitis the mean CRP was significantly higher in PWH compared to HIV-uninfected patients, despite similar clinical features [34]. Further research is needed to ascertain whether immune dysregulation in HIV may result in an exaggerated immune response to COVID-19, resulting in higher CRP levels. We also found that previously reported indices of severity and elevated markers of inflammation on admission [26], were associated with adverse outcomes in HIV patients.

Evidence now suggests that immune dysregulation in PWH with lower CD4 counts could potentially result in an exaggerated immune response and increased risk of severe COVID-19 by reducing the overall SARS-CoV-2 specific CD4 T cells [10, 35]. The impact of HIV on poor outcomes could relate to HIV-related immunosuppression impairing initial viral control thereby allowing higher viral replication which then sets off greater secondary innate response [30]. Some studies have hypothesised that antiretroviral medicines may have some activity against SARS-CoV-2 [36, 37], but despite our high ART coverage, mortality was not lower in PWH, not supporting a clinically meaningful effect.

With widespread and effective ART coverage, PWH are now living longer and are at risk of NCDs [9]. The high prevalence of NCDs in PWH in this study and others [1, 9, 10] highlights the importance of incorporating prevention and management of NCDs at HIV clinics.

There is a paucity of data on the association of TB with outcome of COVID-19 [38]. The first global cohort of COVID-19 patients with current and previous TB, included patients from 8 countries mainly in Europe [20]. This study had a sample size of only 49 patients (of whom 42 had active TB, and 7 had previous TB) with a mortality rate of 12.3% (6/49). Motto et al. subsequently merged this cohort with 20 hospitalised patients and demonstrated that eight out of the 69 patients died (11.6%) [21]. This study concluded that the mortality was higher in older patients with comorbidities and that TB might not be a major determinant of mortality. Our study demonstrated that mortality was higher in PWH compared to those without HIV who were co-infected with TB. While we were able to demonstrate increased odds of death in COVID-19 patients who had active TB on logistic regression analysis, this finding was not duplicated on survival regression analysis, suggesting overestimation of the true measure of association. There was also no association with mortality in those with a prior history of TB. In addition to HIV itself, potential hypotheses for the increased risk of deaths in PWH could be dual lung pathology, thrombo-embolic events, or possibly delayed TB diagnosis and presentation due to COVID-19 restrictions or lockdowns, resulting in patients being unable to access care [39, 40].

Our study has certain limitations, not uncommon with operational data. Laboratory tests were not done in all patients as these were done at the discretion of the treating clinicians. Since this missing data was not random, this may have introduced bias. The true extent of obesity may have been underrepresented since formal BMI measurements were not routinely performed in all cases, due to infection control measures and severity of patient illness. CD4 counts may have been done during the acute admission which may limit interpretation of this variable. Evidence supporting the use of corticosteroids emerged during the study period [23], resulting in changes to treatment protocol. Lack of data regarding steroid prescription may have thus influenced our results. Lack of data regarding the site (pulmonary vs. extrapulmonary) and treatment duration of TB is another limitation which may have provided additional insight about TB and COVID-19 co-infection. This study was conducted early in the pandemic when the original SARS-CoV-2 virus was dominant. Further research is needed to assess the clinical impact of emerging SARS-CoV-2 variants of concern in PWH and TB.

In conclusion, in this population with a high HIV and TB prevalence, we found that older age, male gender and being overweight or obese were independently associated with an increased risk of death in COVID-19 hospital admissions. While HIV and active TB were associated with increased odds of death on logistic regression, this finding was not duplicated on survival regression analysis, suggesting overestimation of the true measure of association. Our findings emphasise the importance of public health measures to curb obesity, to minimise the risk of severe illness and death among these patients. These patient groups should be prioritised in COVID-19 public health responses, including vaccine allocation.

## Data Availability

The datasets used and analysed for this study are available from the corresponding author upon reasonable request.
